# Frequency of arrhythmia symptoms and acceptability of implantable cardiac monitors in Hemodialysis patients

**DOI:** 10.1186/s12882-017-0740-1

**Published:** 2017-10-10

**Authors:** Naya El Hage, Bernard G. Jaar, Alan Cheng, Chloe Knight, Elena Blasco-Colmenares, Luis Gimenez, Eliseo Guallar, Tariq Shafi

**Affiliations:** 1 Western Pennsylvania Hospital, Pittsburgh, PA USA; 2 Division of Nephrology, Department of Medicine, Johns Hopkins University School of Medicine, 301 Mason Lord Drive, Suite 2500, Baltimore, MD 21224-2780 USA; 3 Welch Center for Prevention, Epidemiology and Clinical Research, Johns Hopkins University, Baltimore, MD USA; 4 Nephrology Center of Maryland, Baltimore, MD USA; 5 Division of Cardiology, Department of Medicine, Johns Hopkins University School of Medicine, Baltimore, MD USA; 6 Division of General Internal Medicine, Department of Medicine, Johns Hopkins University School of Medicine, Baltimore, MD USA; 7 Department of Epidemiology, Johns Hopkins Bloomberg School of Public Health, Baltimore, MD USA

## Abstract

**Background:**

Arrhythmia-related complications and sudden death are common in dialysis patients. However, routine cardiac monitoring has so far not been feasible. Miniaturization of implantable cardiac monitors offers a new paradigm for detection and management of arrhythmias in dialysis patients. The goal of our study was to determine the frequency of arrhythmia-related symptoms in hemodialysis patients and to assess their willingness to undergo implantation of a cardiac monitor.

**Methods:**

We conducted a survey of in-center hemodialysis patients at a hemodialysis clinic in Baltimore, Maryland. We assessed the frequency of arrhythmia-related symptoms and willingness to undergo placement of an implantable cardiac monitor (LINQ, Medtronic Inc.).

**Results:**

Forty six patients completed the survey. The mean age of the survey respondents was 59 years and 65% were male. Symptoms were common with 74% (*n* = 34) of participants reporting at least one arrhythmia-related symptom and many [22% (*n* = 10)] had all 3 symptoms. Among the patients with symptoms, 57% (*n* = 26) reported “heart skipping beats, flopping in chest or beating very hard,” 61% (*n* = 28) reported “heart racing (palpitations),” and 37% (*n* = 17) reported feeling that they “passed out or almost passed out.” The majority of the patients felt that the timing of the symptoms was unrelated to dialysis treatments. The acceptability of the monitoring device implantation was high, with 59% (*n* = 20) of patients with symptoms and 50% (*n* = 6) of patients without symptoms willing to consider it. The main reason for not considering the device was not wanting to have an implanted device.

**Conclusion:**

The prevalence of arrhythmia-related symptoms is high in hemodialysis patients and the majority would consider an implantable cardiac monitor if recommended by their physicians. Routine implantation of cardiac monitoring devices to manage arrhythmias in dialysis patients may be feasible and will provide further insights on the leading causes of morbidity and mortality in dialysis patients.

**Electronic supplementary material:**

The online version of this article (10.1186/s12882-017-0740-1) contains supplementary material, which is available to authorized users.

## Background

Patients with end-stage renal disease requiring dialysis have a high risk of morbidity and mortality. The risk of death in the year after dialysis initiation is 20% and median survival is only 3 years [1]. Despite many improvements in general medical care, the 5-year mortality in dialysis patients approaches 65% and has not improved significantly over the last decade [1]. Cardiovascular disease remains the leading cause of death in dialysis patients (50% of all deaths) and the majority of these deaths (25-40% of all deaths) are due to sudden cardiac arrest [1]. To date, no effective strategies to prevent these deaths have been devised.

Patients treated with dialysis also experience an extraordinarily high risk of atrial fibrillation and stroke. The claims-based prevalence of atrial fibrillation is 20%–50%, [2, 3] but the true prevalence is likely much higher as atrial fibrillation episodes are mostly asymptomatic [4–7]. In patients initiating dialysis, the risk of stroke is 20% and more than half of these strokes are classified as cardioembolic or cryptogenic; [8] atrial fibrillation is a likely underlying culprit [9].

A major limitation to the detection and treatment of arrhythmias in dialysis patients has been the lack of our ability to continuously monitor cardiac rhythms. Dialysis units are not equipped with telemetry and continuous home monitoring with Holter devices can only be used for short periods and is not practical for wide-scale use. Prior attempts with larger subcutaneous implanted cardiac monitors have not been successful [10]. A major recent advancement has been the miniaturization of cardiac monitoring technology that is leadless, contains a battery that lasts 3 years, and can be implanted under the skin in less than 60 s. The Reveal LINQ (Medtronic, Mounds View, MN) measures 1.79 in. by 0.29 in. by 0.16 in. and weighs 0.09 oz and is currently indicated for patients at risk for arrhythmia development [11].

The goal of our study was to determine the frequency of arrhythmia symptoms in hemodialysis patients and to assess their willingness to undergo implantable loop recorder implantation for monitoring cardiac arrhythmias.

## Methods

### Study design

The study was approved by the Johns Hopkins Medicine Institutional Review Board (IRB). The IRB considered verbal consent to be adequate as the study only included participant interview without medical record review or longitudinal data collection. We surveyed all patients undergoing in-center hemodialysis at a dialysis clinic in the Baltimore, Maryland in May 2015. We excluded patients with prior pacemaker or defibrillator placement. After obtaining verbal consent to participate in the survey, we administered a brief questionnaire about symptoms of common arrhythmias (Additional file 1: Appendix). We also showed the patients an actual size picture of the Reveal LINQ device, and asked them if they would be willing to have the device implanted if recommended by their doctor. We summarized the survey responses using means and proportions.

## Results

### Baseline characteristics

We approached 97 patients for the survey, 48 consented, of which 2 were ineligible (due to presence of pacemaker or defibrillator) leading to final survey sample of 46 patients. The mean age of the patients was 59 years and 65% were male (Table 1). Approximately 80% of patients in this dialysis clinic are African-American [12]. 15% (*n* = 7) of participants did not have a past history of diabetes, congestive heart failure, stroke, or irregular heartbeat 33% (*n* = 15) had at least one of these problems, 29% (*n* = 13) had two, 17% (*n* = 8) had 3, and 7% (*n* = 3) had all four of the problems.

**Table 1:** Baseline characteristics of the study population

Characteristics	All patients (*n* = 46)
Age, years	59.2 ± 14.2
Male, %	30 (65.2%)
Comorbidities	
Diabetes	27 (58.7%)
Congestive Heart Failure	19 (41.3%)
Stroke	6 (13.0%)
Myocardial Infarction	5 (10.9%)
Irregular heartbeat	20 (43.5%)

### Arrhythmia-related symptoms

Arrhythmia-related Symptoms were common (Table 2). 74% of participants reported at least one arrhythmia-related symptom. Multiple symptoms were common, with 59% (*n* = 27) of the patients reporting two or more symptoms and 22% (*n* = 10) of the patients reporting all 3 symptoms. Among patients with symptoms, 57% (*n* = 26) reported “heart skipping beats, flopping in chest or beating very hard,” 61% (*n* = 28) reported “heart racing (palpitations),” and 37% (*n* = 17) reported feeling that they “passed out or almost passed out.” Only 2 patients reported symptoms occurring all the time; the majority felt that the symptoms occurred intermittently, and most felt that the symptoms’ occurrence was unrelated to dialysis treatments.

**Table 2:** Arrhythmia-related symptoms in hemodialysis patients

		Relationship to hemodialysis (%, among those with symptoms)			
Symptom question	Prevalence	Before	During	After	Unrelated
Have you ever felt your heart was skipping beats, flopping in your chest or beating very hard?	26 (56.5%)	0	0	5 (19.2%)	21 (80.8%)
Have you had any episodes where you felt like you passed out or almost passed out?	17 (36.9%)	0	3 (17.6%)	4 (23.5%)	10 (58.8%)
Have there been times when your heart races (palpitations)?	28 (60.9%)	1 (3.6%)	2 (7.1%)	7 (25.0%)	18 (64.3%)

### Acceptance of LINQ implantation

After simply looking at the actual size picture of the implantable loop recorder (Additional file 1: Appendix) and without further medical counselling, 56% (*n* = 26) of survey respondents were willing to consider it (Table 3). The acceptability rate was 59% (*n* = 20) among the 34 patients with symptoms and 50% (*n* = 6) among the 12 patients without symptoms. The most common reason for refusing device implantation was simply not wanting a device. None of the participants expressed any cosmetic concerns for not wanting the device.

**Table 3:** Acceptability of implantable cardiac monitor

Device acceptability	Symptoms present	Symptoms absent
N (%)	34 (73.9%)	12 (26.1%)
Device Acceptable	20 (58.8%)	6 (50%)
Device Unacceptable - Reason		
Cosmetic	0	0
Concerned about procedure	3 (8.8%)	0
Concerned about complications	2 (5.9%)	2 (16.7%)
Don’t want a device	10 (30%)	3 (25%)

## Discussion

This study of in-center hemodialysis patients from Baltimore, Maryland provides several important observations. Among the 46 participants completing the survey, the prevalence of arrhythmia-related symptoms was high with 74% reporting at least one symptom and 22% reporting all 3 symptoms. Among patients with symptoms, the prevalence of pre-syncope/syncope symptoms was 37% and the prevalence of palpitations/tachyarrhythmia symptoms was approximately 60%. Acceptability of the implantable cardiac monitor was high, with 59% of patients with symptoms and 50% of patients without symptoms willing to consider it. The main reason for not considering the device was not wanting to have an implanted device.

Cardiovascular disease is the leading cause of death in dialysis patients and its prevalence is 20-100 fold higher in dialysis patients compared with the age-matched general population [13]. Almost 25-40% of all deaths in dialysis patients are classified as sudden deaths or deaths related to arrhythmias [1, 14]. Retrospective observational studies suggest a number of risk factors for arrhythmia-related deaths in dialysis patients, including dialysate composition, [15] dialysis-induced rapid fluid and electrolyte shifts, intradialytic hypotension, [16, 17] and marked electrolyte shifts around the long interdialytic interval during the weekend [18, 19]. Atrial fibrillation is also common in dialysis patients and its prevalence (20%-50%) is likely underestimated from claims-based data [2, 3, 20]. Atrial fibrillation, especially undetected paroxysmal atrial fibrillation, could be a major contributing factor to the high incidence of stroke in dialysis patients [8, 21].

Despite the overwhelming burden of arrhythmia-related morbidity and mortality in dialysis patients, there are very few specific interventions to modify this risk. Most of the current dialysis management practices in place have failed to improve this risk over the past decade, suggesting the failure of the one-size-fits-all approach. Risk modification in individual patients cannot occur without knowledge of specific arrhythmias and specific arrhythmias cannot be detected without long-term ambulatory cardiac monitoring. Our study takes an important step in this direction by exploring patient acceptance of such a device. Further work is needed to determine if this approach can be successfully implemented in clinical practice or research settings.

Although the risk of sudden death remains constant after dialysis initiation, it is likely that the arrhythmias causing sudden death in dialysis patients change over time. It is possible that ventricular tachycardia/ventricular fibrillation are more common early after dialysis initiation due to the presence of occult cardiac ischemia combined with intradialytic hypotension and postdialysis metabolic changes such as hypokalemia, hypocalcemia, and metabolic alkalosis. During later years of dialysis, bradyarrhythmias may be more common due to progressive calcification of the conducting system and ongoing use of medications such as β-blockers. Indeed, recent studies in prevalent dialysis patients with implanted cardiac monitors reported that bradyarrhythmias rather than tachyarrhythmias were more common [22, 23].

Knowledge of specific arrhythmias is needed to institute patient-specific management strategies, many of which are standard of care in clinical practice. For example, risk stratification and management of ventricular tachycardia and bradyarrhythmias can follow standard clinical practice guided by a cardiologist. Similarly, atrial fibrillation may require identification of precipitating factors (volume overload in the interdialytic interval and rapid volume removal during dialysis) and stroke prevention by anticoagulation. It is worth noting that the prevalence of atrial fibrillation in dialysis patients is similar or higher than in patients with cryptogenic stroke, a condition where implantable cardiac monitors are considered standard of care [24]. As most atrial fibrillation events are asymptomatic [4–7] they are less likely to be detected without long-term ambulatory cardiac monitoring.

Prevention and treatment of arrhythmias in dialysis patients will require the ability to detect arrhythmias. However, outpatient dialysis units are not equipped with cardiac telemetry monitoring capabilities. Continuous cardiac monitoring in dialysis patients, outside of the hospital setting, has not been feasible either due to the short-term monitoring periods of current external devices (e.g., 24-48 h Holter) or the large size of implantable devices. The large size of the implantable device was likely a limiting factor in a prior study of dialysis patients where 98% of the patients refused to participate in the study and only 8 patients were recruited [10]. These findings formed the rationale for our study to assess the acceptability of a new small and non-conspicuous cardiac monitoring device, the Reveal LINQ.

The Reveal LINQ is implanted subcutaneously over the pericardium via a short procedure that takes about 1 min with an incision that is < 1 cm and closed by Steri-strips without the need for sutures. This device can continuously record surface electrocardiogram of symptomatic episodes for up to 15 min (four 7.5 min episodes, three 10 min episodes, two 15 min episodes). The device data are then transmitted wirelessly to a central monitoring facility. Reveal LINQ has a lithium carbon monofluoride battery with a battery life of 3 years. The device is currently used for patients with unexplained syncope, those with transient symptoms that may suggest cardiac arrhythmias, suspected atrial fibrillation, or cryptogenic stroke, and those at high risk of cardiac arrhythmias. Complications in non-dialysis patients associated with device implantation are minimal and include infection (1.2%), device migration (< 1%), and pain at the insertion site (< 1%) [11].

Several important limitations of our study are also worth noting. First, we surveyed patients in one inner-city dialysis clinic with a majority of African-American patients. Patients from other settings may have a different prevalence of symptoms and acceptability of the device implantation. Second, we only have data on participants that agreed to answer the survey and selection bias could be a factor in these findings. Third, our survey was conducted by study coordinators that are not medically certified in history-taking or patient counseling, and the questions were limited to the survey. It is possible that the prevalence of these symptoms may be higher when this information is obtained by a trained clinician, or lower if the patients attributed non-specific symptoms to one of the questions that was being asked. Fourth, we did not specifically ask the patients if they will be willing to travel outside the dialysis unit to get the device implanted. However, this was implied as no procedures outside of hemodialysis treatment, including access-related procedures, occur in the dialysis unit. It is possible that patients may be less enthusiastic about a procedure that requires additional appointments at a location different from the dialysis unit. Finally, the assumption of the potential benefit of implantable loop recorders in dialysis patients is based on the high risk of arrhythmias and arrhythmic death in dialysis patients combined with the clinical experience and evidence in non-dialysis populations. Such a use of implantable loop recorders in dialysis patients mirrors clinical management of many other conditions such as management of hypertension and diabetes, where management is based on data from non-dialysis populations. Whether existing data on the potential benefits of implantable loop recorders will be sufficient to convince providers to recommend these devices for their dialysis patients and whether the patients will be willing to undergo these procedures in routine clinical practice is an important question that was not addressed by our study and will need future investigation. We believe that our study provides important preliminary data that can be used to pursue these future investigations and management of arrhythmia-related complications, the major cause of morbidity and mortality in dialysis patients.

## Conclusions

In conclusion, we report a high prevalence of arrhythmia-related symptoms and a high acceptability of a new small implantable loop recorder in hemodialysis patients. Routine implantation of these devices to manage arrhythmias and related complications in dialysis patients may be feasible.

## Additional file

Additional file 1: Study Questionnaire: Appendix. Study Questionnaire. (PDF 157 kb)
