# Supplementary material for: Frequency of arrhythmia symptoms and acceptability of implantable cardiac monitors in Hemodialysis patients
Source: BMC Nephrol. 2017 Oct 10;18:309. doi: 10.1186/s12882-017-0740-1 (PMC5635540; doi:10.1186/s12882-017-0740-1)
Supplement: Additional file 1: — Study Questionnaire: Appendix. Study Questionnaire. (PDF 157 kb) [file 12882_2017_740_MOESM1_ESM.pdf]

Subject ID ______________________
Date ___ ___ /___ ___ /___ ___ ___ ___

Introduction:
I am conducting a survey about abnormal heartbeat in dialysis patients and its complications. Your participation is completely voluntary and will take five minutes or less.

Would you be willing to answer questions in this survey? ☐ Yes ☐ No

If yes: Your completion of this survey will serve as your consent to be in this research
.
If no: Thank you for your time.

Screening Question:
Do you have a Pacemaker or Defibrillator:

- ☐ Yes → Not eligible. STOP.
- ☐ No → Continue

General Information
What is your gender/sex? ☐ Male ☐ Female

What is your date of birth? __ __/__ __/__ __ __ __
MM DD YEAR

Past Medical History
Have you been diagnosed with:

- ☐ Diabetes Mellitus
- ☐ Congestive Heart Failure
- ☐ Irregular heartbeat
- ☐ Stroke
- ☐ Myocardial infarction (heart attack)

If yes, have you had:

- ☐ Balloon angioplasty
- ☐ Stent
- ☐ Heart bypass

Subject ID ________________________
Date ___ ___ /___ ___ /___ ___ ___ ___

Have you noticed any of the following <u>since you started dialysis</u>?

Questions	How often?			Relationship to dialysis?			
	All of the time	Sometimes	Never	Before	During	After	Unrelated
1. Have you ever felt your heart was skipping beats, flopping in your chest or beating very hard?	☐	☐	☐	☐	☐	☐	☐
2. Have you had any episodes where you felt like you passed out or almost passed out?	☐	☐	☐	☐	☐	☐	☐
3. Have there been times when your heart races (palpitations)?	☐	☐	☐	☐	☐	☐	☐

4a. (Ask this question if there are symptoms)
If your doctor wanted you to have a monitor placed under the skin to look for abnormalities in heartbeat, will you be willing to consider it?

- The heart monitor looks like this: show monitor or picture (<u>actual size</u>: 1.8”).
- The monitor is FDA approved and takes 1 minute to place under the skin.
- To place the monitor under the skin, a small incision (less than half inch) is required which is this long: (<u>actual size</u>: ———)

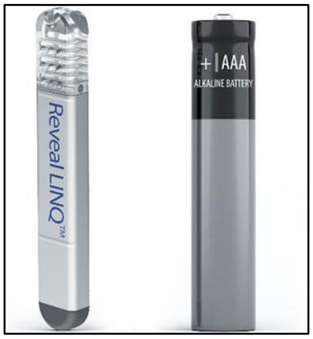

4b. (Ask this question if there are <u>NO</u> symptoms)
Heartbeat abnormalities can cause sudden death in about one out of four dialysis patients. If your doctor wanted you to have a monitor placed under skin to look for abnormalities in heartbeat, will you be willing to consider it?

- The heart monitor looks like this: show monitor or picture (<u>actual</u> size: 1.8”).
- The monitor is FDA approved and takes 1 minute to implant under the skin.
- To place the monitor under the skin, a small incision (less than half inch) is required which is this long: (<u>actual size</u>: ———)

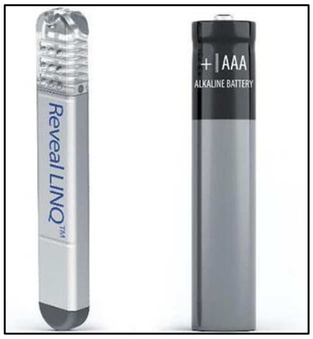

Answer to Question 4: ☐ Yes ☐ No. If No, why not?

☐ Cosmetic reasons.
☐ Concerned about procedure to place device.
☐ Concerned about complications:
If yes, which one: ◻ bleeding ◻ infection ◻ others______________________
☐ Don’t want a device.
☐ Other reasons(please specify): ___________________________

Thank you for answering these questions!
